# The Effect of Exercise on the Skin Content of the Reduced Form of NAD and Its Response to Transient Ischemia and Reperfusion in Highly Trained Athletes

**DOI:** 10.3389/fphys.2019.00600

**Published:** 2019-05-15

**Authors:** Olga Bugaj, Jacek Zieliński, Krzysztof Kusy, Adam Kantanista, Dariusz Wieliński, Przemysław Guzik

**Affiliations:** ^1^Department of Athletics, Strength and Conditioning, Poznań University of Physical Education, Poznań, Poland; ^2^Department of Sport Kinesiology, Poznań University of Physical Education, Poznań, Poland; ^3^Department of Anthropology and Biometry, Poznań University of Physical Education, Poznań, Poland; ^4^Department of Cardiology-Intensive Therapy, Poznań University of Medical Sciences, Poznań, Poland

**Keywords:** NADH, FMSF, incremental exercise test, athletes, mitochondrion

## Abstract

Reduced nicotinamide adenine dinucleotide (NADH) is synthesized in the cellular nucleus, cytoplasm and mitochondria but oxidized into NAD^+^ almost exclusively in mitochondria. Activation of human skin by the 340 nm ultraviolet light triggers natural fluorescence at the light length of 460 nm, which intensity is proportional to the skin NADH content. This phenomenon is used by the Flow Mediated Skin Fluorescence (FMSF) which measures changes in the skin NADH content during transient ischemia and reperfusion. We examined the effects of exercise to exhaustion on the skin changes of NADH in response to 200 s forearm ischemia and reperfusion in 121 highly trained athletes (94 men and 27 women, long-distance running, triathlon, taekwondo, rowing, futsal, sprint running, fencing, and tennis). We found that exercise until exhaustion changes the skin content of NADH, modifies NADH turnover at rest, during ischemia and reperfusion in the most superficial living skin cells. Compared to the pre-exercise, there were significant increases in: mean fluorescence recorded during rest as the baseline value (*B*_mean_) (*p* < 0.001), the maximal fluorescence that increased above the baseline during controlled forearm ischemia (FI_max_) (*p* < 0.001, only in men), the minimal fluorescence after decreasing below the baseline during reperfusion (FR_min_) (*p* < 0.001 men; *p* < 0.01 women) and the difference between *B*_mean_ and FR_min_ (*R*_min_) (*p* < 0.01), and reductions in the difference between FI_max_ and *B*_mean_ (*I*_max_) (*p* < 0.001) and *I*_max_/IR_ampl_ ratio (CI_max_) (*p* < 0.001) after the incremental exercise test. There was no statistical difference between pre- and post-exercise the maximal range of the fluorescence change during ischemia and reperfusion (IR_ampl_)_._ In conclusion, exercise to exhaustion modifies the skin NADH content at rest, during ischemia and reperfusion as well as the magnitude of changes in the NADH caused by ischemia and reperfusion. Our findings suggest that metabolic changes in the skin NADH accompanying exercise extend beyond muscles and affect other cells and organs.

## Introduction

Nicotinamide adenine dinucleotide (NAD) is a cellular coenzyme present in practically all living cells. The two forms of NAD, an oxidized NAD^+^, and reduced NADH can be found in the cell cytosol, nucleus and mitochondria (Dolle et al., 2010; White and Schenk, 2012). Both are crucial in the transfer of electrons between different molecules in reactions catalyzed by oxidoreductases (Mayevsky and Chance, 2007). One of the primary functions of the NAD^+^/NADH coenzymes is their involvement in energy production, which mostly takes places in mitochondria. Therefore, the measurement of these coenzymes is used as a marker of the mitochondrial activity (Mayevsky and Chance, 2007). NAD^+^/NADH are involved in several steps of the citric acid cycle, the transfer of energy and protons between this cycle and oxidative phosphorylation, and the production of adenosine triphosphate (ATP) which is the most essential energy particle (Mayevsky and Chance, 2007; White and Schenk, 2012). NAD has an important function in redox reactions and also serves as a cofactor for many enzymes such as mitochondrial sirtuins and NAD glycohydrolases (Dolle et al., 2013).

The nuclear membrane is permeable for NAD^+^ and NADH through special pores, so the concentration of NAD^+^/NADH is comparable between the nucleus and cytosol. (White and Schenk, 2012). Recent reports indicate that NAD can also penetrate the mitochondrium using an unrecognized NAD (or NADH) transporter (Davila et al., 2018).

While NADH is produced in the nucleus, cytosol, and mitochondria, it is oxidized to NAD^+^ mainly within the mitochondrial electron transport chain (White and Schenk, 2012). It is assumed that mitochondrial NAD^+^/NADH metabolism is similar in all living human cells, e.g., circulating leucocytes, myocytes, liver, brain or skin cells, and comparably affected by blood flow, oxygen and nutrient delivery, as well as inner and external factors or changing conditions, such as physical activity (Green, 1997; Ament and Verkerke, 2009; Mayevsky and Barbiro-Michaely, 2009; White and Schenk, 2012).

It should be noted that the total NAD pool in the body is not constant over a longer time. It is affected by physical activity, diet, and NAD boosters. In addition, NAD pool decreases with aging, as does the sirtuins (NAD-dependent deacetylases) activity that controls almost all cell functions (Kane and Sinclair, 2018; Rajman et al., 2018). The effects of depleted NAD pool may be serious and include several cardiovascular and metabolic disorders (Braidy et al., 2018, Rajman et al., 2018). NAD pool can be reduced due to various factors, e.g., DNA damage, free radicals or excessive ultraviolet radiation. This results in higher activation of poly (ADP-ribose) polymerase (PARP) and increased NAD^+^ turnover and depletion. A chronic immune activation and intensified production of inflammatory cytokines can also occur, leading to increased activity of CD38 protein [a catalyst in cyclic metabolism of ADP-ribose and nicotinic acid adenine dinucleotide phosphate (NAADP)] and, back again, to a decrease in NAD^+^ levels (Braidy et al., 2018).

By contrast, an increased activity of NAD^+^ dependent sirtuins (1–7) has several health benefits related to deceleration of certain aging processes. Sirtuins are located in cell nucleus (SIRT1, 6 – 7), cytoplasm (SIRT1 – 2) and mitochondrium (SIRT 3 – 5). Depending on the location, they support such processes as angiogenesis stimulation or protection against vascular endothelial dysfunction and ischemia-reperfusion damage (Kane and Sinclair, 2018). The intake of NAD^+^ precursors can positively affect cognition function, improve the function of vascular endothelium, act cardioprotective or improve insulin sensitivity and fat free acids oxidation (Rajman et al., 2018).

Initially, studies on the relationship between NADH and physical activity were performed with the use of biopsy samples from working animal muscle (Chance and Connelly, 1957; Edington, 1970; Edington and McCafferty, 1973; Duboc et al., 1988) and in humans (Graham et al., 1978; Sahlin, 1985; Katz and Sahlin, 1987; Sahlin et al., 1987; Graham and Saltin, 1989). Similarly, NADH in skin cells was first studied in animal skin samples (Pappajohn et al., 1972; Palero et al., 2011) and then in humans (Mayevsky and Barbiro-Michaely, 2009; Balu et al., 2013).

Metabolic changes accompanying intensive physical activity, e.g., reduction in pH due to an increase in lactate production (and thus H^+^), modify homeostasis of the whole body, its cells, and organelles, including mitochondria (Green, 1997; Ament and Verkerke, 2009; Kane, 2014). It has been shown that high-intensity exercise modifies the NAD^+^/NADH balance (White and Schenk, 2012). Oxygen is critical for proper mitochondrial energy production. Therefore, a reduction in oxygen content during hypoxia/anoxia observed in the course of ischemia slows down or even stops mitochondrial function, the oxidation of NADH to NAD^+^ and thus the generation of ATP particles (Mayevsky and Rogatsky, 2007). The restoration of oxygen delivery during reperfusion may recover this process. It is unknown, however, whether the alterations in the NAD^+^/NADH balance during ischemia and reperfusion may be affected by metabolic changes induced by intensive exercise.

To explore this problem, we applied the Flow Mediated Skin Fluorescence (FMSF) method which measures the 460 nm fluorescence of NADH in the skin at rest, during controlled ischemia and reperfusion (Piotrowski et al., 2016). This method is based on an optical property of NADH which absorbs light waves in the range of 320 – 380 nm and then emits back the fluorescent light at a longer length of 420 – 480 nm (Mayevsky and Rogatsky, 2007). The intensity of this fluorescence is proportional to the amount of generated NADH. Therefore, changes in fluorescence at a specific light range are monitored to measure the amount of NADH in solutions, cells, tissues, and organs (e.g., brain, liver or skin) (Mayevsky and Rogatsky, 2007). The FMSF is a fluorescence-based method used for the quantification of NADH in a completely non-invasive way and in real time. We hypothesize that intensive exercise to exhaustion influences the contents of NADH in skin epidermal cells, and shifts the NAD^+^/NADH balance toward NADH. Therefore, in this study, we aimed to examine the continuous changes in mitochondrial NADH content at rest, during the controlled 200 s forearm ischemia and the following reperfusion before and immediately after exercise to exhaustion in healthy athletes. Additionally, we analyzed the effects of exercise to exhaustion on the NADH skin content at rest, during ischemia and reperfusion separately for men and women.

## Materials and Methods

### Ethics Statement

The study was designed and performed in agreement with the Declaration of Helsinki. The study protocol and all forms were approved by the Ethics Committee of the Poznań University of Medical Sciences in Poland (no. 1017/16 issued on the 5th October 2016). All athletes participated in this study voluntarily and gave their informed consent.

### Participants

From a group of potential candidates, we excluded athletes who had recent infection or injury with accompanying clinical signs and symptoms such as fever, cough, swelling, or pain. Athletes who were regularly taking any medications for chronic disease not limiting their physical activity (e.g., bronchodilators for asthma, antihistamines for allergy) were also excluded. Only athletes without any medication (except for hormonal contraception) were included.

In Poland, all athletes participating in competitions are required to have a current health certificate every six months; they undergo regular obligatory medical assessment by a physician specializing in sports medicine. Only athletes with a valid health certificate were enrolled.

We recruited 121 healthy highly trained athletes (94 men and 27 women) fulfilling the following inclusion criteria: age in the range between 16 and 40 years, members of Polish National Teams in selected sports disciplines or participants of national and international sports competitions, training at least once a day for at least six days a week. To recruit individuals representing different characteristics of regular training (aerobic, anaerobic, mixed), we selected athletes from the following sports disciplines: long-distance running (*n* = 41), triathlon (*n* = 27), taekwondo (*n* = 25), rowing (*n* = 9), futsal (*n* = 8), sprint running (*n* = 6), fencing (*n* = 4), and tennis (*n* = 1). All participants were examined during the preparatory period of the annual training cycle, i.e., not at the peak of their performance.

### Study Design

The study was conducted in the Human Movement Laboratory of the Department of Athletics Strength and Conditioning, at the Poznań University of Physical Education (Poland). After arriving at the laboratory, we evaluated the health status of each participant. Medical history was obtained and physical examination performed, including brachial blood pressure with the use of the blood pressure monitor Omron M3 (Omron, Japan) in the seated position. Blood pressure was measured twice, at rest before the cardiopulmonary exercise test (CPET) and 2 – 3 min after its completion. The following blood pressure parameters were collected for further analysis: systolic blood pressure (SBP), diastolic blood pressure (DBP), and pulse pressure (PP).

**FIGURE 1:**
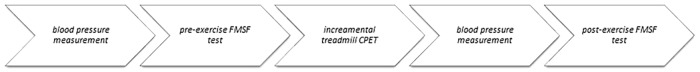
The measuring procedures using the Flow Mediated Skin Fluorescence before and after the cardiopulmonary exercise test.

The majority of participants underwent the treadmill CPET at least once previously. Nevertheless, all athletes were informed about specific requirements and potential risks related to this test. Each participant was asked to avoid any intensive training in the last 48 h preceding the CPET, drinking alcohol for 24 h and coffee or caffeine containing drinks or supplements for 12 h. On the CPET day, participants were allowed to eat a light breakfast. Before the CPET, each participant spent 30 min in the air-conditioned laboratory for acclimatization to its environment. The temperature was kept constant within a range of 19–21°C. Before the CPET and 3–4 min after its completion, the flow-mediated skin fluorescence (FMSF) by the AngioExpert device (Angionika, Poland) was measured in each athlete. Figure 1 summarizes the measuring procedure of FMSF.

### Incremental Cardiopulmonary Exercise Testing

The incremental CPET was performed on a treadmill model 150/50 LC (pluto^®^) with the following sizes of the running surface: length 1500 mm and width 500 mm, (H/P Cosmos Pulsar, Germany). Before the test, all athletes were equipped with a chest strap heart rate monitor (Polar H6 Bluetooth Smart; Polar Electro Oy, Finland) and a properly sized face mask (Cortex Medical, Germany) connected to the MetaMax 3B-R2 mobile spiroergometry device. The CPET started at a speed of 6 km/h for the 4 min warm-up, and then the treadmill speed increased by 2 km/h every 3 min. The treadmill’s incline was set at a constant value of 1%. The CPET was stopped when the athlete reached voluntary exhaustion. After stopping the treadmill, the athlete stood for at least 2 min during recovery and finally was disconnected from the face mask and the Polar chest heart rate monitor. All parameters were measured continuously and then averaged for each breath by the MetaSoft Studio 5.1.0 Software (Cortex Biophysik, Germany). For this study, we used only data on heart rate (HR), estimated maximal heart rate according to the formula “207- 0.7 × age” (Gellish et al., 2007) and the peak or maximal oxygen consumption (VO_2_max) normalized to body mass. VO_2_max was measured only when all of the following three criteria were fulfilled: VO_2_ reached plateau despite a further increase in speed of the treadmill, HR reached at least 95% of age-adjusted HR estimate and respiratory exchange ratio ≥ 1.1 (Edvardsen et al., 2014). In the remaining cases, only the VO_2_ peak value was used for the description of the intensity of exercise.

### NADH Fluorescence

**FIGURE 2:**
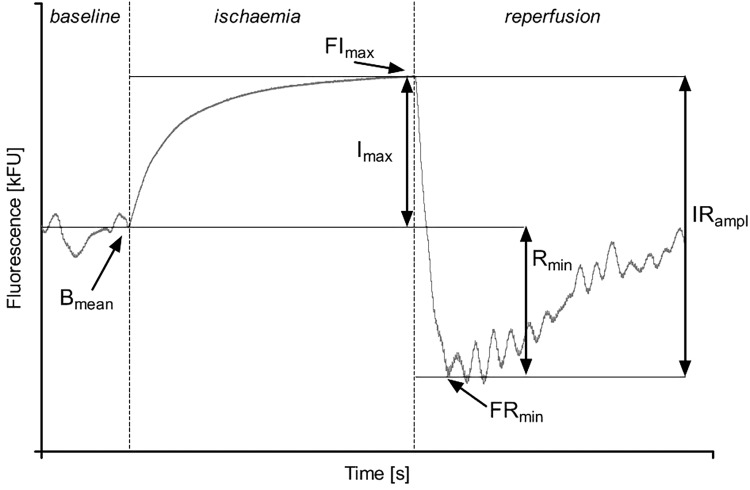
Parameters describing the Flow Mediated Skin Fluorescence: *B*_mean_ – mean of the basal fluorescence; FI_max_ – maximal fluorescence value during ischemia; FR_min_ – the first minimal fluorescence value during reperfusion; *I*_max_ – the net increase of fluorescence over the baseline value during ischemia; IR_ampl_ – the amplitude of fluorescence change during ischemia and reperfusion; *R*_min_ – the net reduction of fluorescence below the baseline value.

The AngioExpert device (AngioExpert, Angionica, Poland) (Piotrowski et al., 2016) was employed to evaluate the 460 nm fluorescence of the skin in response to activation by the 340 nm UV light (Mayevsky and Chance, 1973; Friedli et al., 1982; Mayevsky and Chance, 2007; Mayevsky and Barbiro-Michaely, 2009; Mayevsky et al., 2011; Sibrecht et al., 2017). The wavelength of 340 nm emitted in the FMSF method is only specific to NADH, thus no other substance in the skin can be excited. The 460 nm fluorescence in response to activation takes place mainly in epidermal cells (Dunaev et al., 2015), because the wavelength of the emitted light penetrates and is absorbed up to 0.5 mm deep. The AngioExpert continuously measured the 460 nm fluorescence from the most superficial skin cells in the forearm at rest, then during controlled ischemia triggered by total occlusion of the brachial artery by the brachial blood pressure cuff, and, finally, during reperfusion after deflation of the blood pressure cuff (Piotrowski et al., 2016; Sibrecht et al., 2017) (Figure 2).

To quantify the FMSF response, we used the following parameters (Figure 2) (Sibrecht et al., 2017):

• *B*_mean_ [kFU] – mean fluorescence at 460 nm recorded before ischemia as the baseline value;
• FI_max_ [kFU] – the maximal fluorescence that increased above the baseline during controlled forearm ischemia;
• FR_min_ [kFU] – the minimal fluorescence after decreasing below the baseline during reperfusion;
• *I*_max_ [kFU] – the difference between FI_max_ and *B*_mean_;
• *R*_min_ [kFU] – the difference between *B*_mean_ and FR_min_;
• IR_ampl_ [kFU] – the maximal range of the fluorescence change during ischemia and reperfusion;
• CI_max_ – *I*_max_/IR_ampl_ ratio.

For this study, we continuously measured and recorded skin fluorescence for 2 min before ischemia, then for 200 s of ischemia and finally for 3 min during post-ischemic reperfusion. To obtain total net forearm ischemia, we used a brachial blood pressure cuff placed on the ipsilateral arm and inflated the cuff to 50 mmHg of pressure above the systolic blood pressure of each participant. Such pressure is necessary for temporal, short-lasting compression of the artery and complete cessation of blood flow to the forearm and hand below. The transient forearm ischemia is usually obtained by this procedure in many physiological and clinical studies, for example during testing the flow-mediated arterial vasodilation (Harris et al., 2010; Pahkala et al., 2011; Bailey et al., 2017). The measurements were performed in laboratory conditions in a constant environment according to a strict procedure as described elsewhere (Piotrowski et al., 2016). To avoid any environmental interferences, during the measurement all subjects kept their studied forearm motionless in a special curved form with the fluorescence sensor placed at its bottom. The forearm covered completely the fluorescent sensor to prevent any transmission of external light which might interact with the sensor and fluorescence measurement. Since the fluorescence is monitored and measured continuously with the sampling rate of 25 Hz, the shown signal is a continuous line. Any unexpected sudden departure from this continuity other than caused by a controlled ischemia and reperfusion was considered as an artifact and either not taken to further analysis or instantly repeated, if possible. The repetition was allowed only if the artifacts were present at rest, before any ischemia.

### Statistical Analysis

Continuous data distribution was analyzed using the Kolmogorov–Smirnoff test. Due to normal data distribution, results are presented as mean values and standard deviations (SD). The comparisons between pre- and post-exercise results were made with the paired *t*-test- first for all athletes and then separately for men and women. Only *p* < 0.05 was considered statistically significant. All statistical analyses were made using MedCalc Statistical Software version 18.2.1 (MedCalc Software bvba, Ostend, Belgium^1^; 2018).

## Results

### Baseline Characteristics

The mean age of all participants was around 23 years, their BMI, resting systolic and diastolic blood pressures were normal. The average peak heart rate obtained during the CPET was nearly 189 beats/minute, which corresponded to almost 95% of the estimated maximal HR indicating a very intense (maximal) exercise. Table 1 summarizes baseline data all studied athletes.

**Table 1:** Clinical characteristics of studied athletes.

Parameter:	Mean	SD
Age [years]	23.41	5.32
Training experience [years]	8.15	3.44
BMI [kg/m^2^]	22.29	2.55
SBP at rest [mmHg]	125.88	12.18
DBP at rest [mmHg]	72.20	7.87
PP at rest [mmHg]	53.68	12.26
SBP after CEPT [mmHg]	146.08	22.34
DBP after CEPT [mmHg]	74.42	8.21
PP after CEPT [mmHg]	71.66	24.53
Predicted maximal HR [beats/minute]	190.61	5.32
Peak HR during CPET [beats/minute]	188.88	9.77
Achieved percentage of the predicted maximal HR [%]	99.09	9.53
VO_2_max [ml/min/kg]	60.61	8.03

*BMI, body mass index; CPET, cardiopulmonary exercise test; DBP, diastolic blood pressure; HR, heart rate; PP, pulse pressure; SBP, systolic blood pressure; VO_2_max, maximal oxygen uptake.*

Separate clinical characteristics for participating men and women are shown in Table 2.

**Table 2:** Clinical characteristics of male and female athletes.

	Men		Women	


Parameter:	Mean	SD	Mean	SD
Age [years]	23.88	5.69	21.74	3.35
Training experience [years]	7.71	2.73	7.56	1.48
BMI [kg/m^2^]	22.64	2.57	21.06	2.07
SBP at rest [mmHg]	128.91	11.21	115.30	9.25
DBP at rest [mmHg]	73.01	7.50	69.37	8.63
PP at rest [mmHg]	55.9	12.29	45.93	8.55
SBP after CEPT [mmHg]	148.98	23.32	136.00	14.91
DBP after CEPT [mmHg]	74.18	8.36	75.26	7.76
PP after CEPT [mmHg]	74.8	25.24	60.74	18.43
Predicted maximal HR [beats/minute]	190.28	3.98	191.78	2.34
Peak HR during CPET [beats/minute]	188.96	9.11	190.77	10.37
Achieved percentage of the predicted maximal HR [%]	98.29	11.27	95.84	19.89
VO_2_max [ml/min/kg]	62.25	7.28	54.73	7.96

*BMI, body mass index; CPET, cardiopulmonary exercise test; DBP, diastolic blood pressure; HR, heart rate; PP, pulse pressure; SBP, systolic blood pressure; VO_2_max, maximal oxygen uptake.*

### Effects of Exercise on FMSF

**FIGURE 3:**
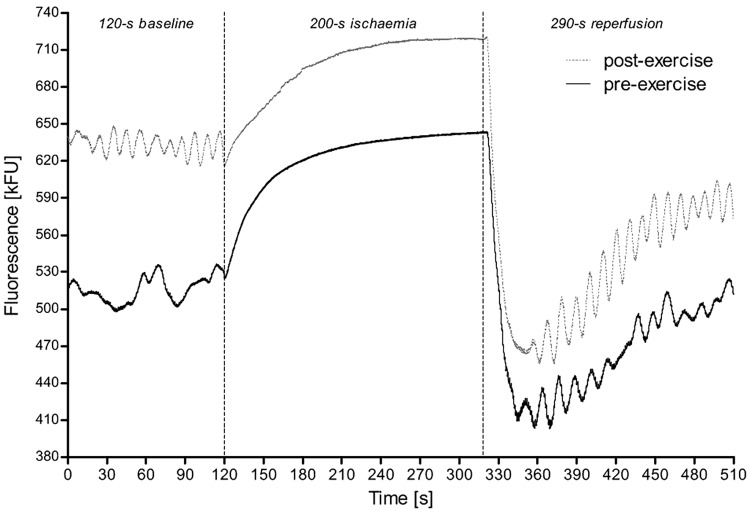
A sample of the FMSF measurement from a 28-year-old female athlete (triathlete) before and after exercise test until exhaustion. The first 2 min correspond to the time before ischemia, which is then followed by the transient 200 s ischemia (gradual increase in the fluorescence) with subsequent reperfusion (sudden drop in the fluorescence). Of note, although in this figure the 290 s reperfusion is shown, for the study we used only the values recorded during the first 180 s.

Figure 3 shows an example of the typical 460 nm fluorescence at baseline, during ischemia and the following reperfusion before and after CPET to exhaustion.

Results of the comparison of the FMSF performed before and after the CPET are shown in Table 3 for all athletes and in Table 4 separately for sportsmen and sportswomen.

**Table 3:** Comparison of the FMSF results acquired before (pre-exercise) and after (post-exercise) the CPET till exhaustion in all studied athletes.

	Pre-exercise		Post-exercise		

Parameter	Mean	SD	Mean	SD	*P*-value
*B*_mean_ [kFU]	404.19	214.31	455.30	256.76	< 0.001
FI_max_ [kFU]	480.98	258.19	514.46	282.99	< 0.001
FR_min_ [kFU]	321.22	168.90	359.07	196.50	< 0.001
*I*_max_ [kFU]	76.79	47.64	59.16	36.33	< 0.001
IR_ampl_ [kFU]	159.76	94.49	155.40	95.46	0.261
*R*_min_ [kFU]	82.97	50.22	96.24	65.98	0.001
CI_max_	0.48	0.08	0.40	0.11	< 0.001

*Averaged data are presented as the mean and standard deviation (SD), and the results of the paired t-test as p-value. B_mean_, mean of the basal fluorescence; CI_max_ – I_max_/IR_ampl_ ratio; FI_max_ – maximal fluorescence value during ischemia; FR_min_ – the first minimal fluorescence value during reperfusion; I_max_ – the net increase of fluorescence over the baseline value during ischemia; IR_ampl_ – the amplitude of fluorescence change between ischemia and reperfusion; R_min_ – the net reduction of fluorescence below the baseline value.*

**Table 4:** Comparison of the FMSF results acquired before (pre-exercise) and after (post-exercise) the CPET till exhaustion in male and female athletes.

	Men					Women				

Parameter	Pre-exercise		Post-exercise		*P*-value	Pre-exercise		Post-exercise		*P*-value
				
	Mean	SD	Mean	SD		Mean	SD	Mean	SD	
*B*_mean_ [kFU]	366.06	198.17	415.34	241.96	< 0.0001	536.95	218.97	594.42	262.69	0.0003
FI_max_ [kFU]	437.77	242.09	473.59	273.02	0.0003	631.43	260.33	656.79	275.53	0.0583
FR_min_ [kFU]	289.41	154.65	327.37	181.78	< 0.0001	431.97	172.41	469.39	209.13	0.0026
*I*_max_ [kFU]	71.71	47.21	58.24	38.70	< 0.001	94.46	45.66	62.36	26.87	0.0003
IR_ampl_ [kFU]	148.36	92.69	146.21	98.57	0.5933	199.46	91.49	187.38	77.03	0.2510
*R*_min_ [kFU]	76.65	49.00	87.97	65.70	0.0028	104.99	49.06	125.02	59.49	0.0025
CI_max_	0.48	0.08	0.42	0.11	< 0.0001	0.47	0.05	0.34	0.09	< 0.0001

*Averaged data are presented as mean and standard deviation (SD), and the results of the paired t-test as p-value. B_mean_, mean of the basal fluorescence; CI_max_, I_max_/IR_ampl_ ratio; FI_max_ – maximal fluorescence value during ischemia; FR_min_ – the first minimal fluorescence value during reperfusion; I_max_ – the net increase of fluorescence over the baseline value during ischemia; IR_mpl_ – the amplitude of fluorescence change between ischemia and reperfusion; R_min_ – the net reduction of fluorescence below the baseline value.*

In all athletes (Table 3), compared to the pre-exercise rest, after the CPET there were significant increases in *B*_mean_, FI_max_, FR_min_ and *R*_min_, and reductions in *I*_max_, CI_max_. No statistical differences between pre- and post-exercise were observed for IR_ampl_.

Both in male and female athletes exercise to exhaustion cause similar changes in the FMSF response. After the CPET there were significant increases in *B*_mean_, FR_min_ and *R*_min_, and reductions in *I*_max_, CI_max_. However, the value of FI_max_ increased significantly only in men but not in women. In both groups, no statistical differences were found for IR_ampl_.

In general, these findings on the skin fluorescence at 460 nm translate to a significant increase in the skin NADH content before and during ischemia, and the following reperfusion after the CPET both in male and female athletes. Additionally, the relative (compared to pre-ischemic baseline) increase in the skin NADH during ischemia (*I*_max_) was significantly lower, and its reduction during reperfusion (*R*_min_) was more substantial after the CPET. Compared to pre-exercise, the contribution of the NADH change during ischemia to the maximal change of its content during both ischemia and reperfusion (CI_max_) was also reduced after the CPET. Changes in *I*_max_, *R*_min_, and CI_max_ were similar in male and female athletes.

## Discussion

We have found that exercise until exhaustion caused a significant elevation of the 460 nm skin fluorescence at baseline (*B*_mean_), during controlled ischemia (FI_max_) and reperfusion (FR_min_) compared to the pre-exercise values. However, the observed post-exercise increase of the 460 nm fluorescence during ischemia (*I*_max_) was smaller than before exercise. In contrast, the magnitude of the fluorescence drop during the reperfusion (*R*_min_) was larger after exercise than before exercise. Additionally, the relative contribution of the ischemia-induced increase in fluorescence to the whole change in fluorescence during ischemia and reperfusion (CI_max_) was reduced after exercise. Except FI_max_, all descriptors of the FMSF changed, similarly, in male and female athletes. The lack of significant increase (borderline *p* = 0.0583) in FI_max_ in women appears to be caused by a much smaller size group of studied female athletes. Altogether, our findings suggest that exercise to exhaustion causes a significant increment in the NADH content in mitochondria. However, the ischemia-related rise in NADH is smaller whereas the reperfusion-related oxidation of NADH is more intense after than before exercise. These effects of exercise appear to be similar both in sportsmen and sportswomen.

There is some balance between the reduced (NADH) and oxidized (NAD^+^) forms of NAD. Although the 460 nm skin fluorescence measures only NADH, its values also reflect indirectly NAD^+^ as the total amount of NAD (i.e., combined amount of NADH and NAD^+^) seems to be rather stable at a relatively short period (a few minutes) necessary to perform the CPET. In other words, the NADH amount changes at the cost of the NAD^+^ content, and vice versa.

Previous animal and human studies showed that the amounts of NADH and NAD^+^ and the NAD^+^/NADH ratio change during ischemia and reperfusion. Palero et al. (2011) studied the effects of hypoxia on NADH in mice keratinocytes. They observed that NADH accumulated in the complex I of the electron transport chain and, in contrast to common knowledge, keratinocytes metabolism is not only anaerobic since it strongly depends on the oxygen delivery by blood in the underneath skin layers. Balu et al. (2013) used a clinical multiphoton microscope, applied arterial occlusion to human keratinocytes and found that, during ischemia, the NADH content increased in the cells near the basal layer of the skin (although not in the keratinocytes closest to the skin surface). Our study shows similar results obtained in the living human skin in real time using a completely non-invasive physiological model. Because the fluorescence emitted by skin derives only from the most superficial zone of depth up to 0.1 mm (Balu et al., 2013), it means that skin cells at this depth have a vivid metabolism of NADH, and react in a dynamic way to ischemia-triggered hypoxia and then to re-oxygenation during reperfusion.

Other studies have shown that the amount of NADH and/or NAD^+^ also changes during exercise (White and Schenk, 2012). Depending on the intensity, an exercise may be accompanied by an aerobic, mixed aerobic-anaerobic or purely anaerobic metabolism. During aerobic or oxygen-dependent metabolism, various nutrients are turned into energy in mitochondria. When oxygen supplies are lower than needed, anaerobic processes start in the cytoplasm and with time predominate over aerobic energy production.

With progressing hypoxia until anoxia, the cellular metabolism switches to entirely anaerobic processes which are accompanied by various changes in the intra- and extracellular milieu (White and Schenk, 2012). The most classic consequences within the cells and between the intra- and extracellular spaces are changes in the concentration of H^+^, lactate, ammonium, adenosine, and ions such as sodium, potassium, calcium, magnesium, and chlorides. Many metabolic effects of ischemia resemble those observed during anaerobic exercise. In both conditions, when oxygen is depleted, the NAD^+^ particles are partially restored from NADH in the cytosol by reduction of pyruvate acid to lactic acid, with an accumulation of the latter as the by-product (Robergs et al., 2004; Finsterer, 2012; Kane, 2014). However, this way of energy generation is not as efficient as NADH oxidation in the mitochondrial electron transport chain during the aerobic effort – less ATP is made at the cost of higher nutrient usage and due to the accumulation of lactate.

Exercise until exhaustion alters the NAD^+^/NADH ratio and, most probably, the rate of NADH oxidation to NAD^+^. However, data on NAD^+^ during exercise are sparse (White and Schenk, 2012). Whereas some researchers observed an exercise-induced increase in NAD^+^ (Chance and Connelly, 1957; Jobsis and Stainsby, 1968), others found no change or reduction in its amount (Duboc et al., 1988; White and Schenk, 2012). Duboc et al. (1988) reported that it is NADH that increases during exercise. Other authors noticed that untrained animals had a higher concentration of mitochondrial NAD^+^ than trained ones (Edington, 1970; Edington and McCafferty, 1973). Sahlin (1985) studied NADH in human muscle during short-lasting intensive exercise and found that at least 95% of the cellular NADH comes from mitochondria. This author also observed that NADH increases within 2 min after exercise initiation and it remains elevated during and immediately after the exercise completion. Additionally, 10 min is required for the NADH to return to baseline level after the exercise. Next, Sahlin et al. (1987) studied NADH in muscle samples taken after the exercise at the level of 40, 75, and 100% of VO_2_max. The light exercise at the intensity of 40% VO_2_max was accompanied by a decrease in the NADH content. In contrast, more intense exercise at the level of 75 and 100% VO_2_max caused a significant increase in the NADH content. Graham et al. found that the NAD^+^ content in human muscles decreased after moderate (75% of VO_2_max) and high intensity (100% of VO_2_max) exercise (Graham et al., 1978; Graham and Saltin, 1989). Stabilization of metabolic processes requires some time and depends on several factors, e.g., the active recovery (Menzies et al., 2010) and the level of physical performance (Ravier et al., 2006). Few minutes after intensive exercise is not enough for full metabolic recovery, including the complete removal of an excess of H^+^ or full restoration of the aerobic metabolism. It is plausible that one of the main limiting factors for a rapid scavenging of the accumulated NADH during exercise is the system of the malate-aspartate shuttle (O’Donnell et al., 2004; White and Schenk, 2012; Satrústegui and Bak, 2015) that transfers H^+^ from cytosol to the mitochondrial matrix and enables NAD oxidation and reduction. It appears that the turnover rate of the mitochondrial shuttles is rather constant, or even reduced under specific conditions, and thus some time is required to transfer the excess of H^+^ from cytosol into mitochondria (O’Donnell et al., 2004). Simultaneously, intact NAD can penetrate the mitochondrium using a still unrecognized NAD or NADH transporter (Davila et al., 2018). We assume, however, that this transporter has a limited capacity.

So far, NADH metabolism has been studied either during ischemia/reperfusion (hypoxia/normoxia) or in various exercise models, mostly with the use of invasive methods. To the best of our knowledge, none of the studies has ever shown the combined effect of exercise and ischemia-reperfusion on NADH, additionally using a completely non-invasive approach for measurement of NADH. In our study, the absolute fluorescence values of *B*_mean_, FI_max_, and FR_min_ were significantly higher after exercise than before it. This increase in fluorescence might be the net effect of the accumulation of the pre-exercise and newly produced NADH during the maximal exercise, particularly during the anaerobic part of the effort. The additional potential explanation is that the altered post-exercise metabolic conditions slowed the function of the MAS and also other shuttles, transporting H^+^ to electron transport chain. The MAS activity can also be attenuated and even stopped by an increase of Ca^2+^ concentration in the cytoplasm (O’Donnell et al., 2004; Contreras and Satrústegui, 2009; Satrústegui and Bak, 2015), i.e., a common consequence of an anaerobic effort.

Post-exercise reduction of the *I*_max_ below the pre-exercise level is an interesting finding. The *I*_max_ corresponds to the amount of NADH that is directly generated during skin ischemia. We speculate that an excess of H^+^ and electrons generated during the anaerobic part of the exercise saturated a substantial part of the NAD^+^ and turned it into NADH (an increase of *B*_mean_ and FI_max_). However, since the total amount of NAD in the form of NADH and NAD^+^ is more or less constant in the cells in a short time frame, then less NAD^+^ particles become available for the reduction to NADH during the post-exercise ischemia. It might also be possible that there are some intracellular protective mechanisms preventing an endless intracellular accumulation of NADH as in our model of addictive effects of exercise and ischemia. At the same time, an increase of the *R*_min_ value suggests that in the early phase of post-exercise reperfusion more NADH is oxidized to NAD^+^ or some mechanisms promoting more rapid regeneration of NAD^+^ are activated. Further, it appears that after exercise the limitation of the NADH increase during ischemia and the enhancement of the NADH oxidation to NAD^+^ during reperfusion are comparable to those observed at rest. It shows that the responses of the NAD^+^/NADH to ischemia and reperfusion are set at the specific individual range different for each person. Additionally, it suggests that there is a relative resistance of cells to produce an unlimited amount of NADH during such a dramatic metabolic challenge as maximal exercise.

Since the 460 nm fluorescence of the skin increases after exercise, it is the NADH that raises during and after maximal exercise. However, we measured the skin and not muscle NADH metabolism. Due to a significant contribution of skin in thermoregulation, it is plausible that the NADH changes in the skin might be not the same as in the muscles. With increasing intensity and duration of exercise, skin blood flow becomes relatively reduced as most of the blood is redirected toward working muscles, however, immediately after the exercise, skin blood vessels dilatation takes place. Despite a sudden influx of blood to the skin, and connected oxygen supply, other metabolic processes do not allow for quick restoration of homeostasis. It is possible that during exercise to exhaustion, there is some overlap and additive effect of metabolic changes in the muscles caused by anaerobic processes and relative skin ischemia due to some constriction of skin arteries. Nevertheless, it is assumed that mitochondrial function is generally the same in different types of cells, and thus changes observed in the mitochondria of skin cells should be, at least to some extent, similar to changes in mitochondria of the working muscles myocytes. In our study, we did not take into consideration the effect of factors acting over a longer period of time, such as sirtuins, PARP and CD38, because the measurement using FMSF method was of short duration and, consequently, the total NAD pool remained unchanged. We also did not investigate the effect of exercise training on change in NADH, but only the response to a single exercise bout (test until exhaustion).

Limitations of the study must be recognized. To study the NADH metabolism we used the continuous measurement of the autofluorescence by skin cells activated by the ultraviolet light. The application of the 460 nm fluorescence as a way of measuring NADH has been known for years. The group of Mayevsky made a series of seminal studies on the practical use of measuring the NADH content in skin cells or superficial cells of other organs (Mayevsky and Chance, 2007; Mayevsky et al., 2011) in humans. The FMSF method is further, although very specific, development of this approach, which has been proposed by Piotrowski et al. (2016). In the FMSF method, the 460 nm skin fluorescence is not only measured at rest but also during a dynamic metabolic challenge caused by the transient and controlled ischemia, and the following reperfusion. A potential limitation is also a heterogenic group of athletes. All of them are elite athletes at the national or international level representing different sports and adapted to different training types: endurance, speed, strength, speed-power, etc. However, we used the identical CPET test with the same goal – to stop it after exhaustion which is very individual and subjective. In this way, we were able to collect a wide range of VO_2_max, resting and peak values of heart rate in healthy and physically active people. Next, our study was somewhat more exploratory than explanatory. We had a single and simple aim – to see whether maximal exercise-induced alterations in cell metabolism may influence the NADH metabolism of skin cells during ischemia and reperfusion. We were more interested in the combined effect of both provocations. Our data show some relevant alterations in the NADH during both processes and suggest that non-invasive FMSF method and the model of skin cells can be applied in the studies on physical exercise. It should also be mentioned that one of the limitations of the 460 nm fluorescence method is skin color of studied athletes. Although our participants came from the Caucasian ethnic group, there were significant differences in skin coloration which, even in the same person, can be modified for example by sunbathing or tattooing. To limit these effects, we used paired statistical tests to compare the same parameters before and after exercise, and in this way, each person was compared with himself or herself. No measurement of NADH in the blood appears to be yet another limitation of our study. However, NADH is mainly present intracellularly, and it is an unstable and highly reactant particle (Singhal and Zhang, 2006). Moreover, NADH is degraded to nicotinamide by the extracellular NAD(P) nucleosidases (Johnson, 1984; O’Reilly and Niven, 2003). Nicotinamide is a final end-product of degradation of NADH and NAD^+^ but also NADPH and NADP^+^, so measurement of its changes is not specific for NADH only. There is also another technical problem – during complete brachial artery occlusion used for the controlled ischemia, there is no blood flow what makes a collection of blood samples impossible. The blood can be sampled only before ischemia and during the post-ischemic reperfusion but, as we show, the NADH concentration decreases within a couple of seconds after the restoration of the blood flow, i.e., before the blood might be even sampled. Nevertheless, we believe that studying the NADH concentration in the blood before and after ischemia at different conditions like rest and post-exercise deserves further exploration. Although all studied participants were elite athletes, this group was quite heterogenic and representing different sorts of sports. However, we did not find any significant differences between the included sports disciplines and obtained results. Since the number of athletes representing different sports disciplines varied from 1 to 41, we cannot exclude that there are some potential differences, which might be revealed if larger groups of athletes were studied. Finally, we have not measured the skin NAD^+^ content as this form of NAD does not emit the fluorescent light at the length of 460 nm. Any conclusions on NAD^+^ come from the assumption that the total amount of NAD in its reduced (NADH) and oxidized (NAD^+^) forms is rather stable over short time. If so, then any change in the NADH is at the cost of NAD^+^ and vice versa. In consequence, although NAD^+^ was not measured, some conclusions on its changes may be drawn from studying the alterations of the NADH content.

### Perspectives or Practical Applications

There are some instant potential consequences of our findings. First, the exercise-induced changes in NADH reflect mitochondrial function and a significant part of energy metabolism – these aspects are of great importance in the sports physiology. Second, it is highly probable that the observed post-exercise changes in the skin NADH content reflect metabolic alterations in the myocytes, and, if so, we assume that the FMSF might be used as a non-invasive and an *in vivo* approximate model of the working muscles. In this way, studying the effects of training on NADH metabolism appears to be simpler and more available now. Third, the anaerobic muscle metabolism during exercise to exhaustion is similar to tissue hypoxia which can be observed in different clinical conditions like chronic heart failure, cardiogenic shock, anemia, acute limb ischemia or high mountains environment. Any pharmacological or non-pharmacological intervention designed to improve oxygen delivery or tissue tolerance to hypoxia might be thus easier to study with the FMSF method. Similarly, any intervention designed to change the total amount of NAD content might be easily studied with this method. We are, however, aware, that these consequences are only some speculations as we have not tested them. Therefore they require future studies.

## Conclusion

This is the first study which non-invasively evaluated the NADH skin content in human superficial skin cells in highly trained athletes. Until now methods used to evaluate NADH level, and therefore mitochondrial function, were not easily accessible. Metabolic changes, elicited by exercise to exhaustion, modify the skin NADH metabolism at rest, during ischemia and reperfusion in the most superficial living skin cells. Immediately after exercise, there is a shift of the baseline fluorescence of NADH in the skin cells toward higher values. The absolute NADH amount increases during post-exercise ischemia and reperfusion, compared to resting condition. However, compared to resting conditions, the relative rise in the NADH is significantly lower during ischemia, whereas the relative reduction in the NADH during reperfusion increases. These changes in the NADH metabolism during ischemia and reperfusion before and after exercise to exhaustion appear to be similar in male and female athletes.

The observed alterations in the NADH amount and its balance with NAD^+^ during ischemia and reperfusion are strongly dependent on metabolic conditions, which are significantly modified by exercise to exhaustion, and last for the next few minutes after it. The intensification of NADH fluorescence in living skin cells suggests that metabolic changes in NADH accompanying exercise extend beyond muscles and affect other cells and organs.

## Ethics Statement

This study was carried out in accordance with the recommendations of the Ethics Committee of the Poznań University of Medical Sciences in Poland with written informed consent from all subjects. All subjects gave written informed consent in accordance with the Declaration of Helsinki. The protocol was approved by the Ethics Committee of the Poznań University of Medical Sciences in Poland (no. 1017/16 issued on the 5th October 2016).

## Author Contributions

OB designed the study, recruited and tested participants, analyzed and interpreted data, performed literature research, prepared the manuscript. JZ contributed to the study conception and design, recruited and tested participants, analyzed and interpreted data, performed literature research, prepared and revised the manuscript. KK contributed to the study conception and design, recruited and tested participants, analyzed and interpreted data, revised the manuscript. AK recruited and tested participants, revised the manuscript. DW tested participants, contributed to data analysis and interpretation. PG contributed to the study conception and design, performed data and statistical analysis and interpreted results, performed literature research, prepared and revised the manuscript.

## Conflict of Interest Statement

The authors declare that the research was conducted in the absence of any commercial or financial relationships that could be construed as a potential conflict of interest.

## Footnotes

1 http://www.medcalc.org
